# Supplementary material for: Mapping the planet’s critical areas for biodiversity and nature’s contributions to people
Source: Nat Commun. 2024 Jan 10;15:261. doi: 10.1038/s41467-023-43832-9 (PMC10781687; doi:10.1038/s41467-023-43832-9)
Supplement: Supplementary file 1 — Supplementary Materials [file 41467_2023_43832_MOESM1_ESM.pdf]

SUPPLEMENTARY INFORMATION

Mapping the planet's critical areas for biodiversity and nature's contributions to people

Rachel A. Neugarten, Rebecca Chaplin-Kramer, Richard P. Sharp, Richard Schuster, Matthew Strimas-Mackey, Patrick R. Roehrdanz, Mark Mulligan, Arnout van Soesbergen, David Hole, Christina M. Kennedy, James R. Oakleaf, Justin A. Johnson, Joseph Kiesecker, Stephen Polasky, Jeffrey O. Hanson, Amanda D. Rodewald

Correspondence to: ran63@cornell.edu

This file includes:

Supplementary Methods
Supplementary Figures 1 to 7
Supplementary Tables 1 to 6

Supplementary Methods

Species targets

As described in the Methods, "Spatial optimization" section, we followed previous studies which established targets based on species' habitat size, with the goal of ensuring that both restricted-range and wide-ranging species are represented[1–4]. We set targets as follows (also see Supplementary Table 4): we assigned a 100% threshold to species with less than 1,000 km^2 of suitable habitat (2,391 species of amphibians, 1,024 birds, 680 mammals, and 1,264 reptiles), a 10% threshold to species with more than 250,000 km^2 of suitable habitat (695 amphibians, 5,600 birds, 1,758 mammals, and 589 reptiles), and log-linearly interpolated thresholds for species with intermediate amounts of suitable habitat (2,872 amphibians, 6,296 birds, 2,607 mammals, and 2,168 reptiles; migratory bird species were assigned targets for each seasonal distribution separately). We also assigned a cap of 1,000,000 km^2 for species with a large amount of suitable habitat (>10,000,000 km^2) (six amphibians, 148 birds, 57 mammals, and six reptiles). These targets should be considered minimum representation targets as they do not account for habitat connectivity, ecological intactness[5], species traits[6], evolutionary processes, ecosystem representation[7], genetic diversity, or other important dimensions of biodiversity.

Urban Pressure Index

We created the Urban Pressure Index (UPI) based on global urban growth probabilities for 31 years at approximately the same resolution as the Development Potential Indices (DPIs) (30 arc-seconds, ~1-km) and was based on the SLEUTH urban growth model, which accounts for slope, land cover, excluded regions (i.e., protected areas, water bodies), urban land cover, transportation, and hill shade and calibrated based on the historical distribution of global population from LandScan[8].

To produce an UPI similar to the Development Potential Indices (DPIs) used for other sectors, we removed all currently designated urban areas[9] and existing (non-urban) built-up areas[10]. We then summed urban growth probability values across the 31-year time interval, resulting in values ranging from 1 (1% probability of expansion in 2050) to 3100 (100% probability of expansion for all 31 years). Given the right-skewed distribution of these data (skewness = 4.3619), we log transformed all urban cell values and scaled the data to 0-1 values using min-max normalization.

Because UPI was derived from urban expansion probabilities based on population growth projections, which were more restrictive than the DPIs (e.g., excluded suitable areas like flat land, near roads and existing urban areas once demand was met), we binned the range of values represented in the UPI into either "high" or "very high" categories when combined with other DPI classes within our development pressure index.

Development Pressure

To create our development pressure index, we also used Development Potential Indices (DPIs), because they provide the most globally consistent and detailed data on the relative suitability of lands for future development expansion by agriculture, mining, oil and gas, and renewable

energy sectors. For a more complete discussion of the assumptions and limitations of the DPIs, see[11]. The high and very high DPI classes used in our study were validated as having a higher likelihood of development expansion over other DPI classes[11], as well as to reflect general patterns of potential expansion under business-as-usual growth projections[12]. Accurately predicting locations of future development expansion, however, requires a number of assumptions and a level of precision that are not currently available with global data. For example, governmental actions (e.g., environmental regulations, incentives, tax breaks), market changes and technological advancements were not accounted for in the DPI, again because of a lack of consistent global data. Given the frequency of policy and market changes, variations across administrative units, and the effort required to maintain such a database, incorporating all feasibility factors was beyond the scope of this analysis, but as these data become more consistently available globally, doing so will improve future work.

Projected tree cover loss to 2029 and projected land cover conversion to 2050

The Development Potential Indices we used model the gradient of land suitability for multiple development sectors and does not predict land use change and conversion events. To explore the differences in results based our approach relative to other land cover vulnerability models, we overlaid our maps of prioritized areas with projected tree cover loss to 2029[13] and vulnerability to land cover conversion (cropland and urban expansion) to 2050 from Esri, Clark Labs, and the European Space Agency Climate Change Initiative[14]. Data for first product is available for download: https://zenodo.org/records/3237796[15]. The second product is available for download (https://www.arcgis.com/home/item.html?id=645c280931ac486cadb92c828eac09e3) but, to our knowledge, has not been published in the peer-reviewed literature. Also, this product focuses on agriculture and urban expansion, and is thus limited on the sector drivers it considers. The tree-cover loss product focuses only on a single habitat type (forest). Thus we analyzed both products here only for illustrative purposes. We focused on areas with more than 50% probability of tree cover loss[13] or conversion[14]. Results are shown in Supplementary Fig. 5.

Comparison to previous work

The spatial optimizations included here differ from previous work[16] in several key ways. First, because of the inclusion of biodiversity targets, the optimizations were run globally, rather than for each country, as it is more efficient (in terms of land area required) to achieve species representation targets at a global scale rather than within countries. In order to be consistent with data on terrestrial species, the current analysis focuses on the ten NCP that are relevant for terrestrial habitats, including water-related NCP provided by terrestrial systems (flood regulation, water quality regulation), the onshore component of coastal protection, and nearshore mangrove carbon storage (within 10km of the coast). Offshore marine NCP (marine fish catch, coral reef tourism, the offshore component of coastal protection) were not included, in contrast to[16]. Because the focus of the current analysis is on achieving global biodiversity and climate targets simultaneously, vulnerable ecosystem carbon storage (an NCP with global benefits) was included in all optimization scenarios, rather than in a parallel analysis as was done in[16]. Due to the large number of species and constraints from using traditional computational resources, the spatial resolution of the optimization in the current analysis is 10 km, rather than 2 km as in[16]. This coarser resolution resulted in larger land areas to achieve targets (see Supplementary Fig. 7,

Supplementary Table 6); the difference in resolution was addressed by masking prioritization results to natural and semi-natural habitat data at 2 km[17].

Supplementary Figures

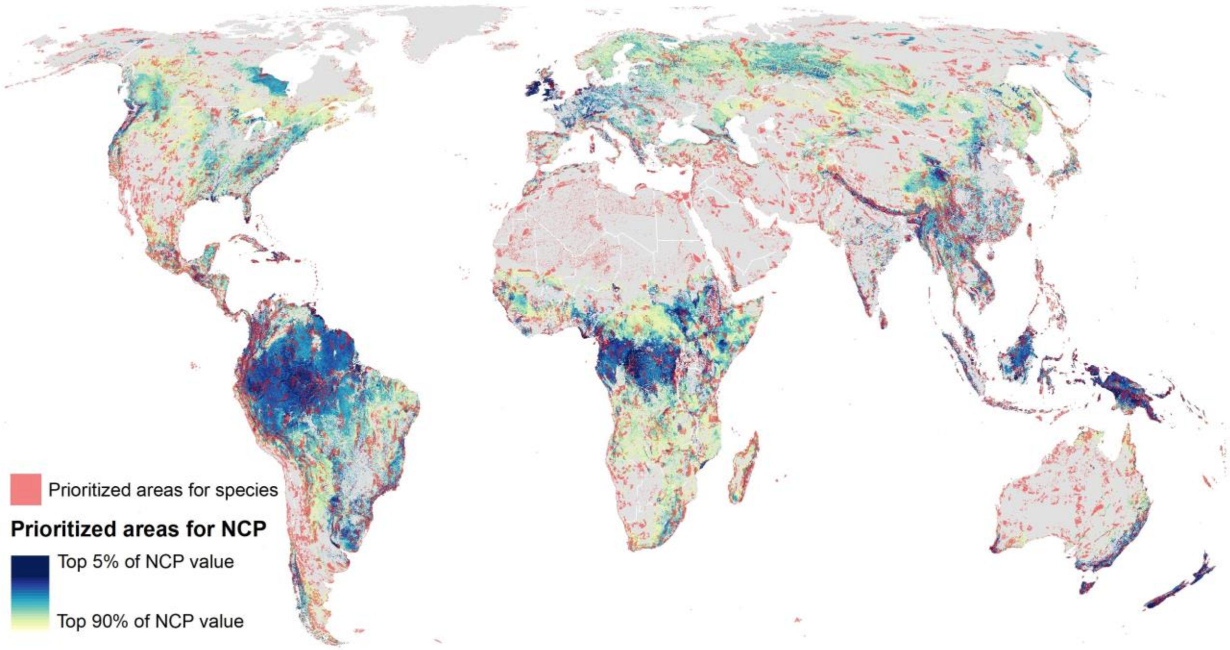

Supplementary Figure 1. Prioritized areas for NCP overlaid with prioritized areas for species. Prioritized areas for NCP (5%-90% of current levels, dark blue to light yellow) overlaid with areas required to achieve minimum species representation targets (in red), optimized separately.

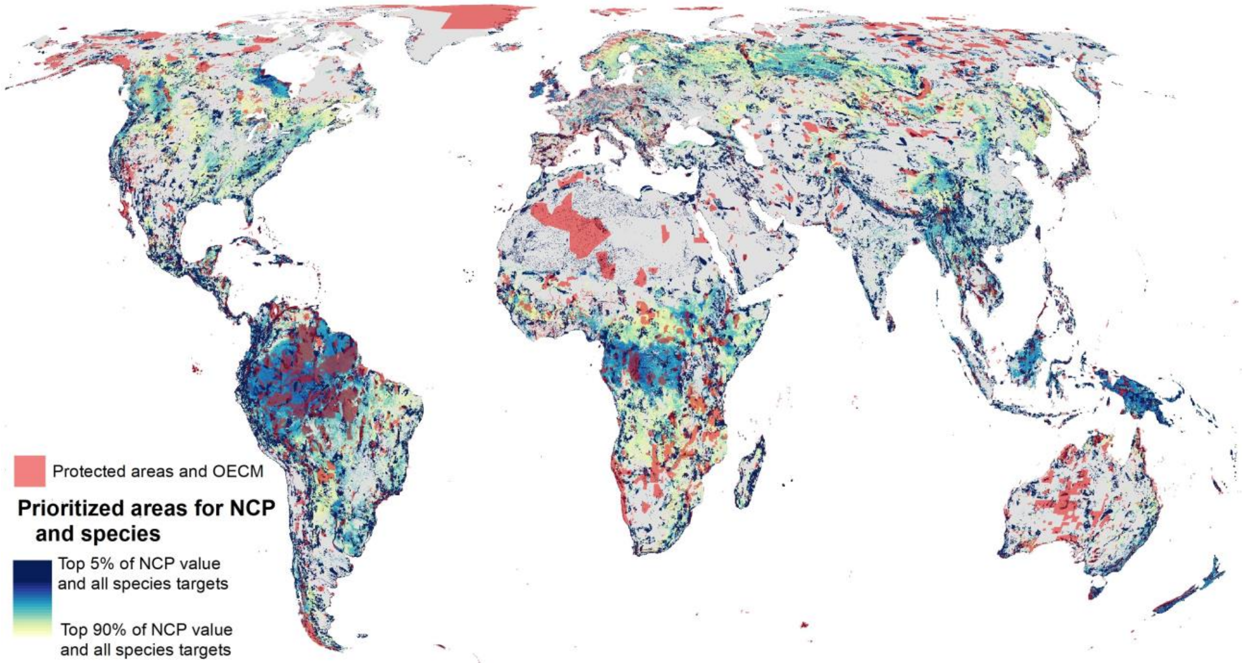

Supplementary Figure 2. Prioritized areas for NCP and species overlaid with terrestrial protected areas and Other Effective area-based Conservation Measures (OECMs)

Protected areas and OECM[18] (shown in red) currently represent 18% of areas prioritized for nature's contributions to people (90% of NCP) and biodiversity.

a

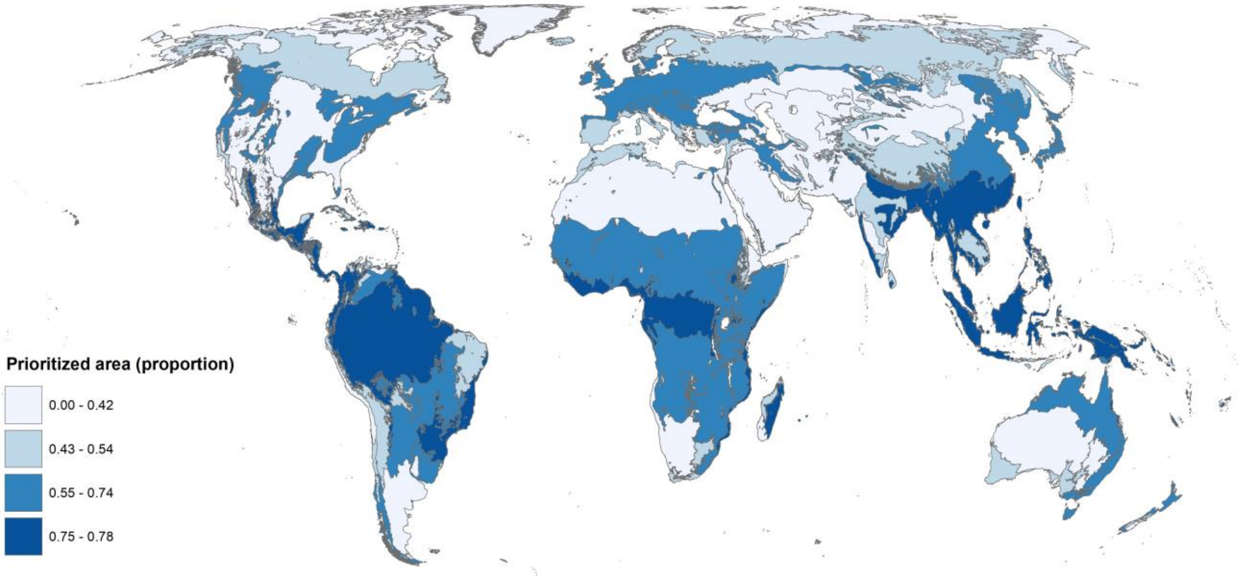

b

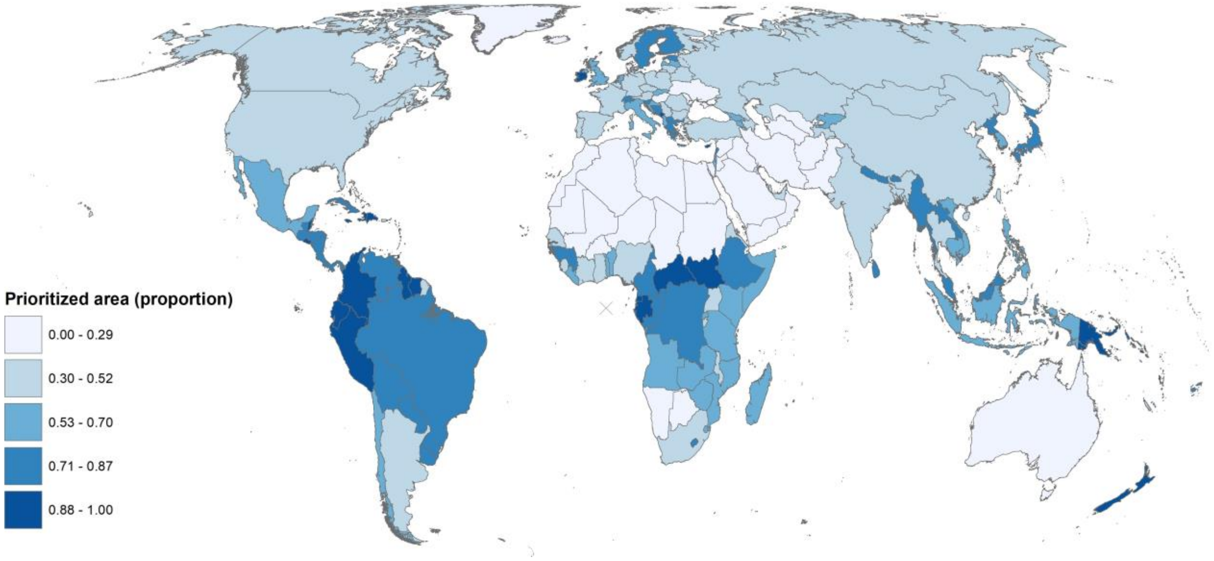

Supplementary Figure 3. Proportion of a) biomes and b) countries with prioritized areas for NCP (90%) and species

Darker blue colors indicate a larger proportion of each biome or country contains prioritized areas. Habitat types (biomes) with a large proportion of prioritized areas include Tropical & Subtropical Moist Broadleaf Forests (78%), Tropical & Subtropical Coniferous Forests (76%), Mangroves (76%), Temperate Conifer Forests (74%), Flooded Grasslands & Savannas (67%), and Temperate Broadleaf & Mixed Forests (57%). Countries with at least 95% of their land area in prioritized areas include: (in South America): Suriname and Guyana; (North America): Antigua and Barbuda, Barbados, Jamaica, Dominica, Saint Lucia, Saint Vincent and the Grenadines; (Oceania): Nauru, Samoa, and New Zealand; (Africa): Central African Republic, Equatorial Guinea, Comoros, and Sao Tome and Principe. See Supplementary Data 1 for complete results.

a

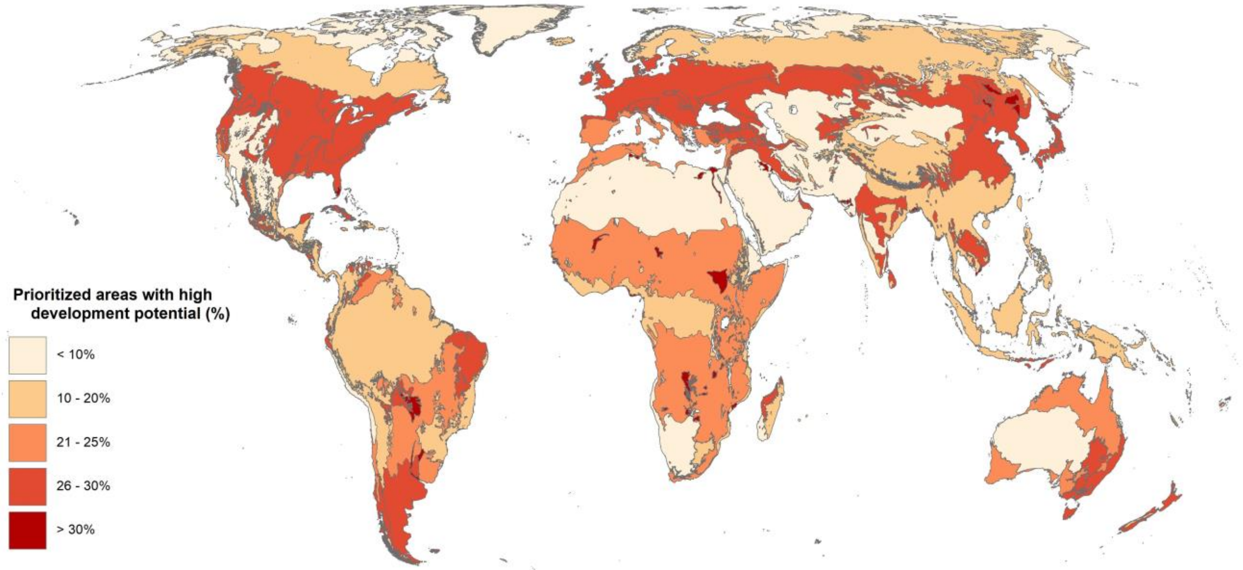

b

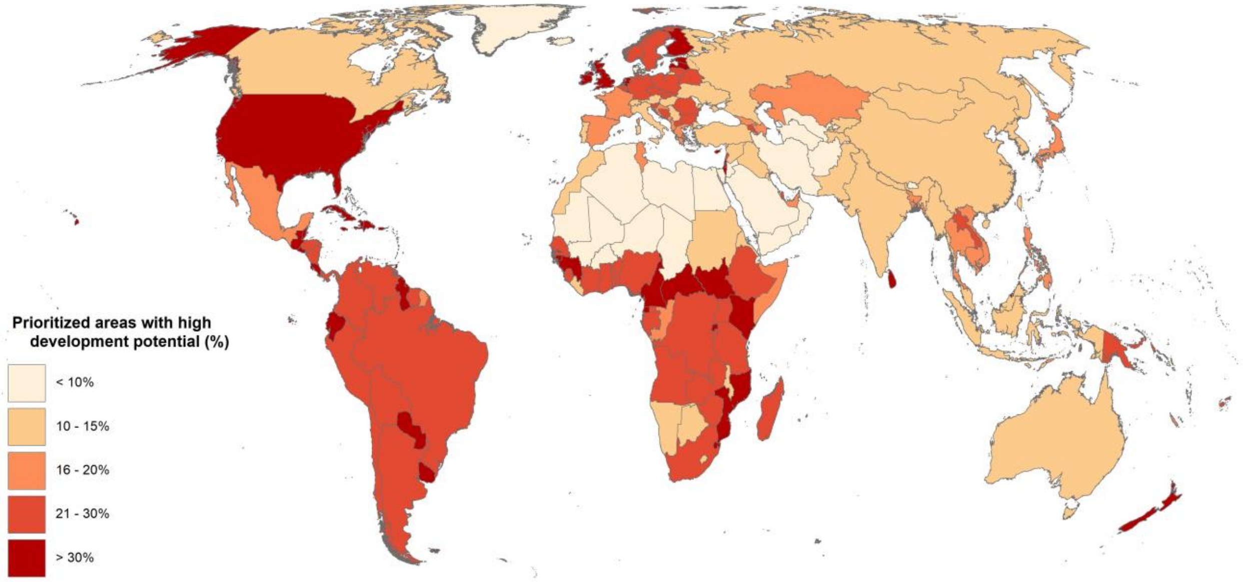

Supplementary Figure 4. Proportion of a) biomes and b) countries with prioritized areas for NCP (90%) and species that also have high development potential

Darker red colors indicate a larger proportion of each biome or country contains prioritized areas that also have high development potential. Major habitat types (biomes) containing extensive prioritized areas that also have high suitability for development include mangroves (32%), temperate broadleaf and mixed forests (30%), and flooded grasslands and savannas (30%). Countries with large proportions of prioritized areas that also have high development potential (>40% of their area) include: (in Africa): Gambia, South Sudan; (Europe): Ireland, Cyprus, Estonia, Netherlands; (North America): Jamaica, Barbados, Haiti; (Asia): Sri Lanka, Bahrain, (South America): Trinidad and Tobago, Uruguay, and Ecuador. See Supplementary Data 2 for complete results.

a

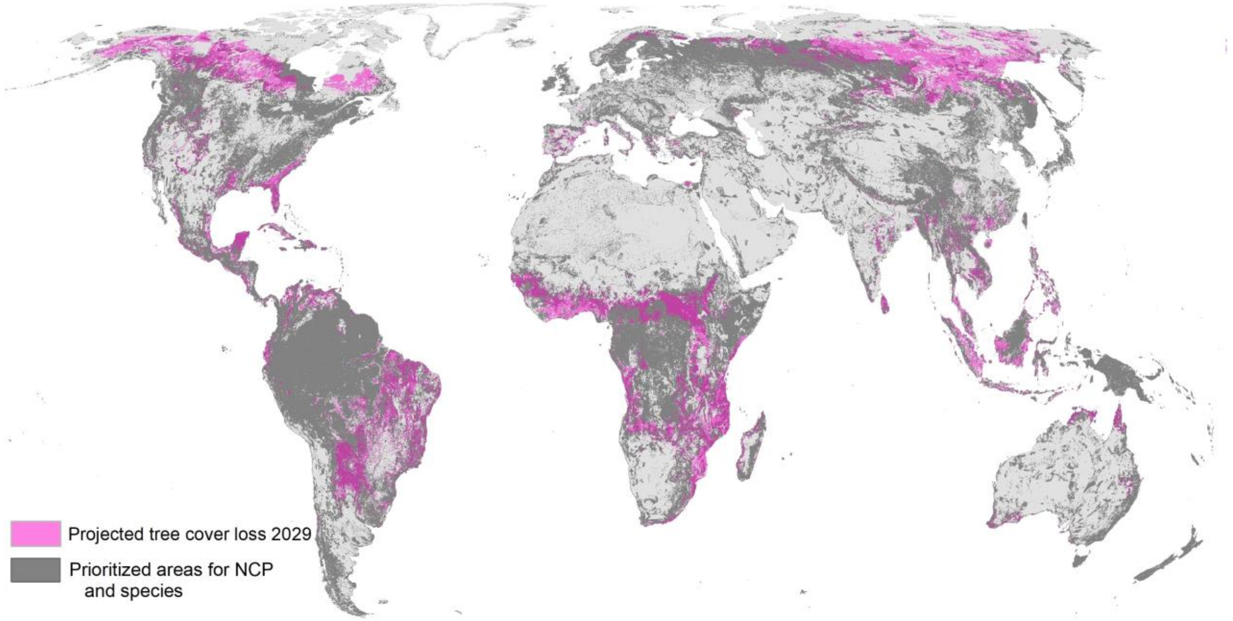

b

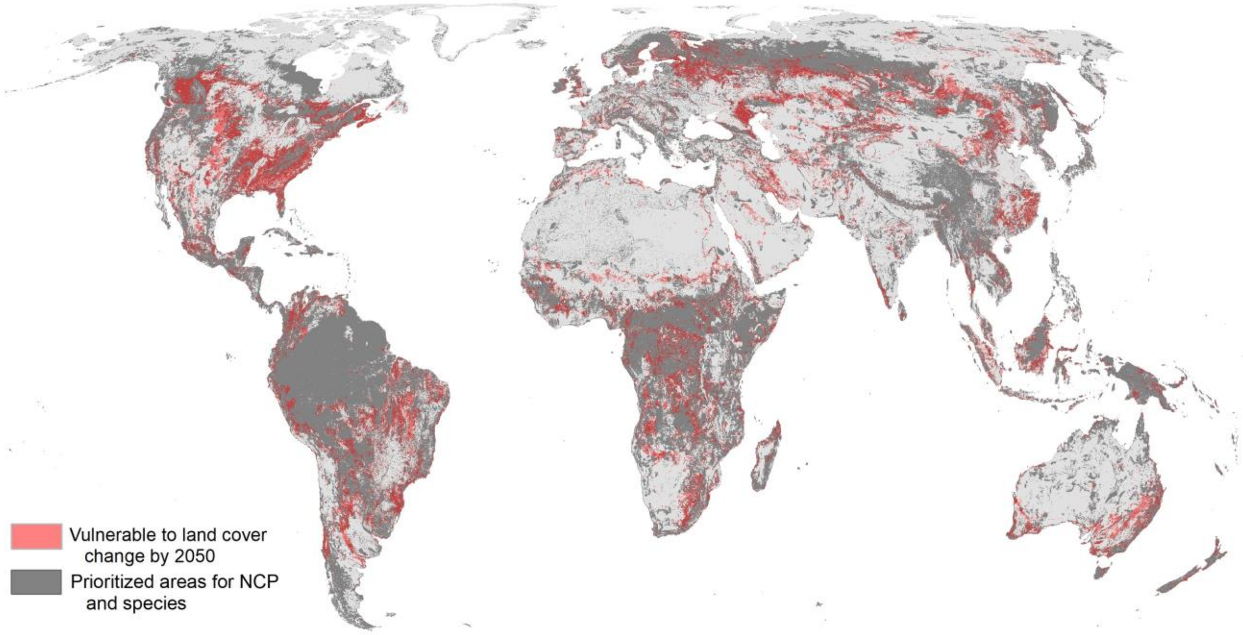

Supplementary Figure 5. Overlap of prioritized areas for NCP and species with global maps of projected tree cover loss to 2029 and projected land conversion to 2050

a) Overlap of prioritized areas for NCP and species (dark gray) with projected tree cover loss to 2029[13], areas with >50% probability of tree cover loss shown in pink, and b) areas vulnerable to land cover change by 2050[14] (areas with >50% probability of land conversion shown in red). Prioritized areas (dark gray) are defined as natural and semi-natural habitats providing 90% of NCP and achieving minimum species representation targets. According to these estimates, 14.6% of prioritized areas may lose tree cover by 2029, and 18.8% of prioritized areas are vulnerable to land cover change by 2050.

a

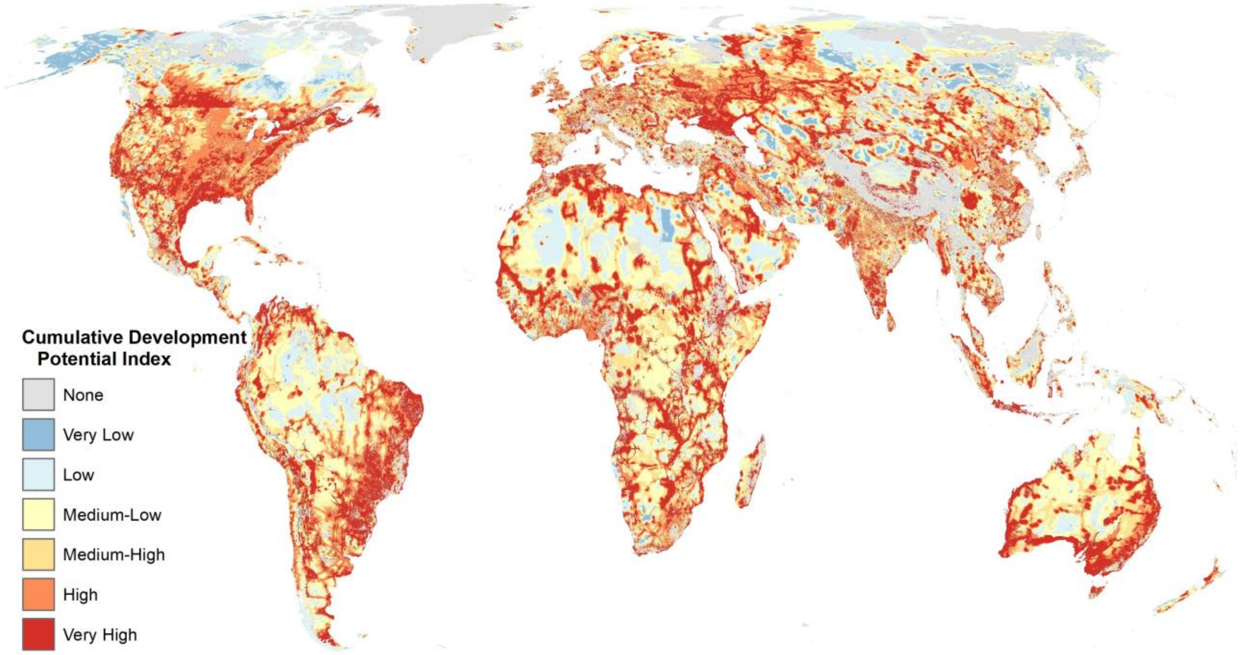

b

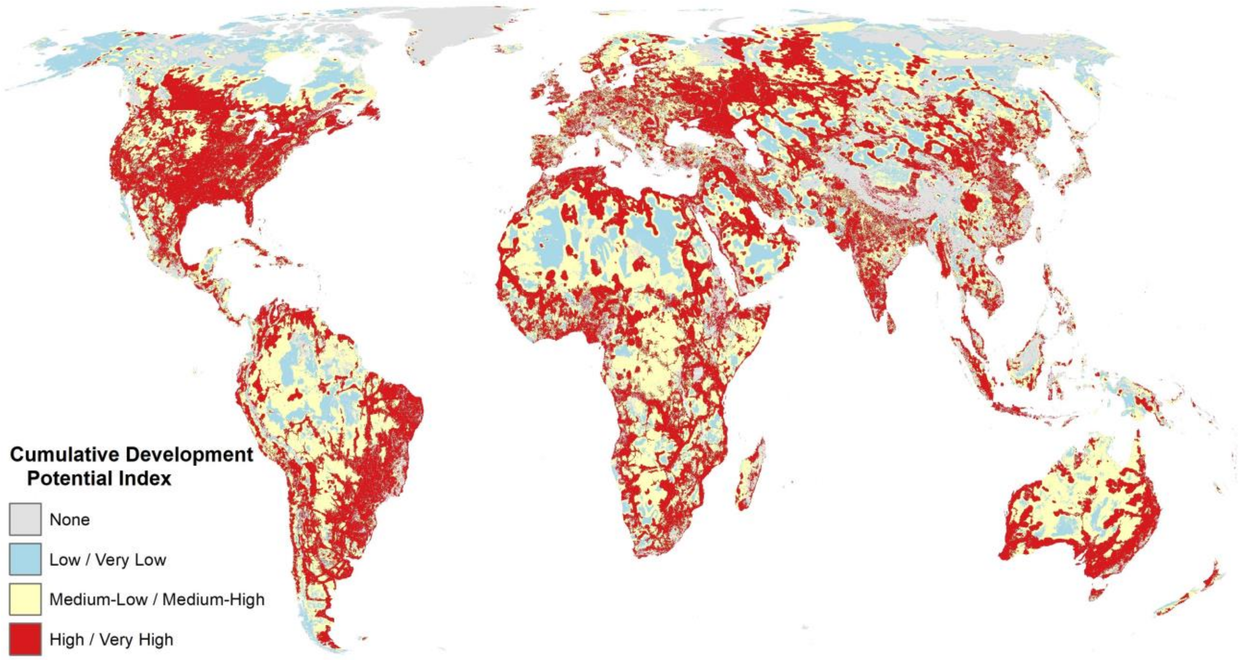

Supplementary Figure 6. Cumulative Development Potential Index

a) Global development potential for renewable energy, oil and gas, mining, agriculture, and urban sectors b) grouped into low, medium, and high development potential. Areas with no or low development potential (blue), areas with medium-low or medium-high development potential (yellow), and areas with high or very high development potential (red). Data available from: https://tnc.box.com/s/cyu1w0c14h8fhl1ln3s01rbj8ickas0t

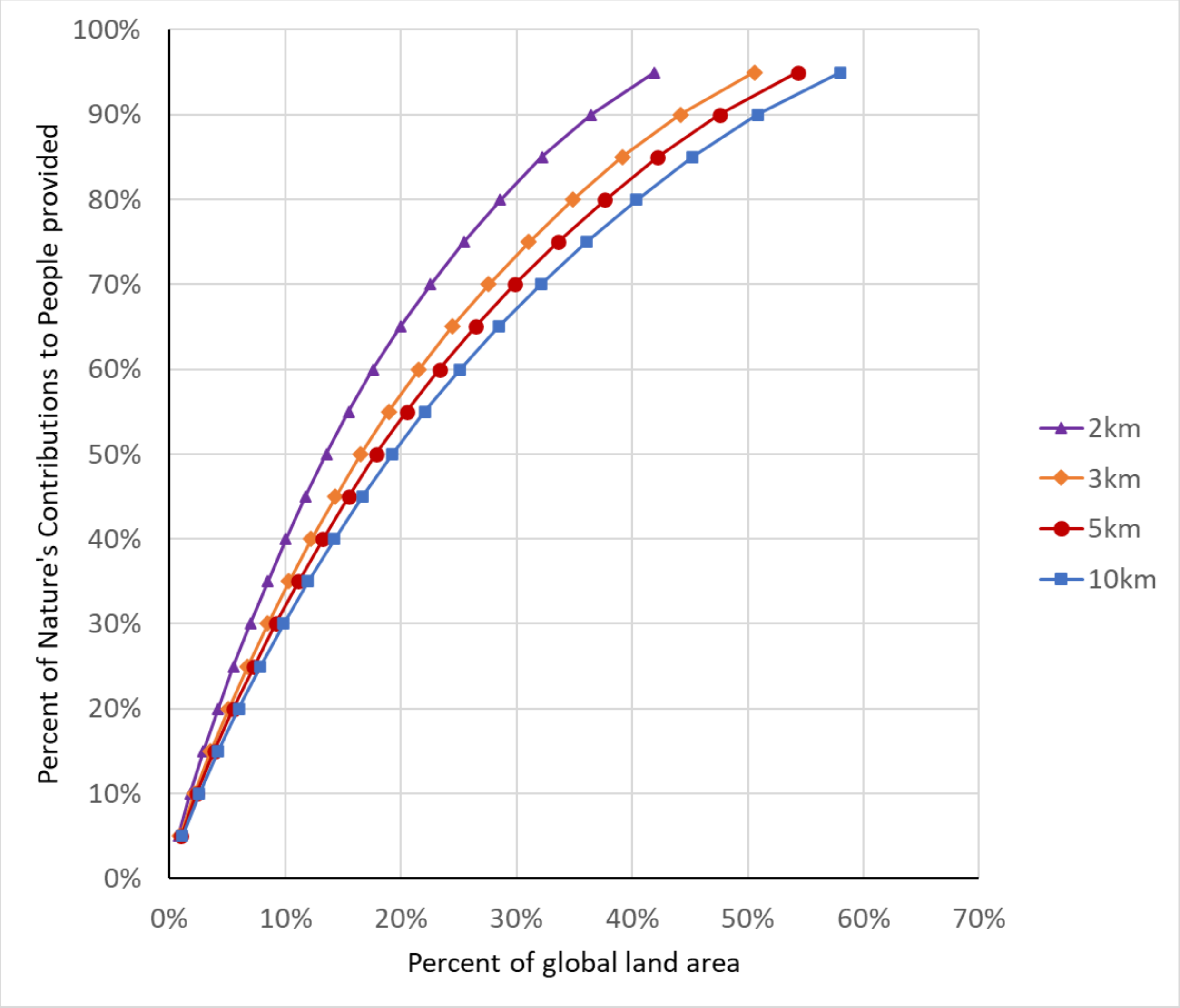

Supplementary Figure 7. Percent of land area required to provide different levels of NCP at different spatial resolutions

Results of prioritization scenarios for NCP (only) with targets ranging from 5% to 95% across all ten NCP. Different symbols represent solutions at different spatial resolutions: 10 km (blue squares), 5 km (red circles), 3 km (orange diamonds), and 2km (purple triangles.)

Supplementary Tables

Supplementary Table 1. List of nature's contributions to people (NCP) included in this analysis "Source" indicates the source of the original data or model documentation, but all datasets were updated (or newly generated) at a global scale by the authors. All NCP are attributed to the natural and semi-natural land cover classes providing the benefit (Supplementary Table 3). "Original resolution" indicates the spatial resolution of the data provided for analysis, but all data were resampled to 10 km for the spatial optimizations. See details in Chaplin-Kramer et al.[16]

Nature's Contribution to People	Source	Units	Original resolution
Nitrogen retention for water quality regulation	Modeled using InVEST, Chaplin-Kramer et al. (2022)[16,19]	Kg/ha nitrogen retained multiplied by number of people downstream	10 arc-sec (~300 m)
Sediment retention for water quality regulation	Modeled using InVEST, Chaplin-Kramer et al. (2022)[16,19]	Tonnes/ha sediment retained multiplied by number of people downstream	10 arc-sec (~300 m)
Crop pollination contribution to nutrition production	Modeled using InVEST, Chaplin-Kramer et al. (2022)[16,19]	"Average people fed equivalents"; average of pollination-derived energy (calories), folate, and vitamin A production divided by RDI.	10 arc-sec (~300 m)
Fodder production for livestock	Modeled using Co$ting Nature[20], Chaplin-Kramer et al. (2022)[16,19]	Index (0-1) of dry matter productivity utilized by livestock	5 arc-min (~10 km)
Timber production (commercial and domestic)	Modeled using Co$ting Nature[20], Chaplin-Kramer et al. (2022)[16,19]	Index (0-1) of accessible timber harvest for commercial & domestic use (optimized separately)	5 arc-min (~10 km)
Fuel wood production	Modeled using Co$ting Nature[20], Chaplin-Kramer et al. (2022)[16,19]	Index (0-1) of fuel wood accessible to local rural communities based on production and access	5 arc-min (~10 km)
Flood regulation	Modeled using WaterWorld[21,22],	Index (0-1) of hydrologically influential "green" (canopy, soil, wetland) water storage	5 arc-min (~10 km)

	Chaplin-Kramer et al. (2022)[16,19]	multiplied by the number of people on downstream floodplains.	
Access to nature (habitat within one hour of rural and urban populations)	Chaplin-Kramer et al. (2022)[16,19]	Count of people within one hour travel time of natural and semi-natural habitat	10 arc-sec (~300 m)
Vulnerable ecosystem carbon storage	Noon et al. 2022[23,24]	Tonnes of carbon/ha (for terrestrial ecosystems, soils, and mangroves). Data available from: https://zenodo.org/records/4091029[24]	1 arc-sec (~30 m)
Coastal risk reduction	Modeled using InVEST, Chaplin-Kramer et al. (2022)[16,19]	Unitless risk reduction index multiplied by number of people within protective distance	10 arc-sec (~300 m)

Supplementary Table 2. Additional data included in the analysis

Name	Description	Source	Resolution
Biodiversity	"Area of Habitat" (AOH) for 26,709 terrestrial vertebrate species	Patrick Roehrdanz, Conservation International, based on IUCN Red List data. Methods described in Brooks et al.[1]	NA (polygon shapefiles)
Protected areas	Protected areas from World Database of Protected Areas (see below)	Jeffrey Hanson, Carleton University, based on WDPA data. UNEP-WCMC and IUCN, [18] www.protectedplanet.net	NA (polygon shapefiles)
Development potential	Global development potential for agriculture, renewable energy, oil and gas, mining, agriculture, and urban sectors	Christina Kennedy and James Oakleaf, The Nature Conservancy. Oakleaf et al.[11,25] Data available from: https://zenodo.org/records/7853188	1 km
Country and continent boundaries	Country and continent boundaries	Esri.[26] Data available from: https://www.arcgis.com/home/item.	NA (polygon shapefiles)

		html?id=d974d9c6bc924ae0a2ffea0a46d71e3d	
Biome boundaries	Terrestrial biomes (groups of ecoregions)	Dinerstein et al.[27]. Data available from: https://ecoregions2017.appspot.com/	NA (polygon shapefiles)
Land cover	Masks for NCP layers included all land cover classes from ESA 2015 except for cropland, mosaic cropland, urban areas, bare areas, water bodies, permanent snow & ice	ESA Climate Change Initiative - Land Cover project [17] Data available from: https://maps.elie.ucl.ac.be/CCI/viewer/download.php	10 arc-sec (~300 m)
Projected tree cover loss	Projected tree cover loss to 2029 (transition potential >50%)	Hewson et al. 2019.[13,15] Data available from: https://zenodo.org/records/3237796	1 km
Areas vulnerable to land cover change	Converted lands 2018 to 2050 (conversion probability >50%)	Esri, Clark Labs, and the European Space Agency Climate Change Initiative[14]<br>Data available from: https://www.arcgis.com/home/item.html?id=645c280931ac486cadb92c828eac09e3	300 m

Supplementary Table 3. European Space Agency land cover classes to which NCP were masked ESA Land Cover – Climate Change Initiative [17]

ID	Description	Grazing: herbaceous	Timber: forests	Fuelwood: woody	All other terrestrial NCP: natural / semi-natural
10	Cropland, rainfed				
11	Cropland, rainfed, herbaceous cover				
12	Cropland, rainfed, tree or shrub cover				
20	Cropland, irrigated or post-flooding				
30	Mosaic cropland (>50%) / natural vegetation (tree, shrub, herbaceous cover)(<50%)	X	X	X	X
40	Mosaic natural vegetation (tree, shrub, herbaceous cover) (>50%) / cropland(<50%)	X	X	X	X
50	Tree cover, broadleaved, evergreen, closed to open (>15%)		X	X	X

ID	Description	Grazing: herbaceous	Timber: forests	Fuelwood: woody	All other terrestrial NCP: natural / semi-natural
60-62	Tree cover, broadleaved, deciduous, closed to open (>15%)		X	X	X
70-72	Tree cover, needle leaved, evergreen, closed to open (>15%)		X	X	X
80-82	Tree cover, needle leaved, deciduous, closed to open (>15%)		X	X	X
90	Tree cover, mixed leaf type (broadleaved and needle leaved)		X	X	X
100	Mosaic tree and shrub (>50%) / herbaceous cover (<50%)	X	X	X	X
110	Mosaic herbaceous cover (>50%) / tree and shrub (<50%)	X	X	X	X
120-122	Shrubland	X		X	X
130	Grassland	X			X
140	Lichens and mosses	X			X
150	Sparse vegetation (tree, shrub, herbaceous cover) (<15%)	X	X	X	X
151	Sparse tree (<15%)	X	X	X	X
152	Sparse shrub (<15%)	X		X	X
153	Sparse herbaceous cover (<15%)	X			X
160	Tree cover, flooded, fresh or brackish water		X	X	X
170	Tree cover, flooded, saline water		X	X	X
180	Shrub/ herbaceous cover, flooded, fresh/saline/brackish water	X		X	X
190	Urban areas				
200-202	Bare areas				
210	Water bodies				
220	Permanent snow and ice				

Supplementary Table 4**.** Count of species in each taxonomic group falling into each Area of Habitat (AOH) category and associated minimum representation target used in optimizations

Area of Habitat (AOH) size	Minimum representation target used in optimization (all scenarios)	Count of species	Taxonomic group	Count of species
< 1,000 km^2	100%	5359	Amphibians	2391
			Birds*	1024
			Mammals	680
			Reptiles	1264
1,000 – 250,000 km^2	(Log-linear interpolated target from 10-100%)	13943	Amphibians	2872
			Birds*	6296
			Mammals	2607
			Reptiles	2168

250,000 - 10,000,000 km^2	10%	8642	Amphibians	695
			Birds*	5600
			Mammals	1758
			Reptiles	589
>10,000,000 km^2	10% or 1,000,000 km^2, whichever is smaller	217	Amphibians	6
			Birds*	148
			Mammals	57
			Reptiles	6

Supplementary Table 5. Results from all prioritization scenarios
First column: target levels of NCP, ranging from 5%-95% of current levels of all 10 NCP. Second column: global land area required to provide target levels of NCP. Third column: area required to provide target levels of NCP and achieve minimum species representation targets (species targets are consistent across all scenarios, see main text). Fourth column: area required to provide target levels of NCP and achieve minimum species targets, with current PAs and OECM areas "locked in" to prioritization results. Land area percentages exclude Antarctica. NA values indicate that a given NCP target is exceeded once other objectives (e.g. representing species, or locking in protected areas and OECM areas) are achieved. For example, 16% of global land area is required to achieve minimum species representation targets, and those areas already provide 20% of NCP, so lower NCP targets are not applicable.

Target (% of current levels of NCP provided)	NCP	NCP and species	NCP and species, protected and OECM areas "locked in"
5%	1%	NA	NA
10%	2%	NA	NA
15%	3%	NA	NA
20%	4%	16%	NA
25%	6%	18%	NA
30%	7%	19%	NA
35%	8%	21%	28%
40%	10%	22%	29%
45%	12%	23%	30%
50%	14%	25%	31%
55%	15%	27%	33%
60%	18%	28%	34%
65%	20%	30%	36%
70%	23%	32%	38%
75%	25%	35%	40%
80%	29%	37%	43%
85%	32%	40%	46%
**90%**	**36%**	**44%**	**49%**
95%	42%	49%	53%

Supplementary Table 6. Percent of land area required to provide different levels of NCP (5%-95% of current levels) at different spatial resolutions

NCP provided	Spatial resolution			
	2km	3km	5km	10km
5%	1%	1%	1%	1%
10%	2%	2%	2%	3%
15%	3%	4%	4%	4%
20%	4%	5%	6%	6%
25%	6%	7%	7%	8%
30%	7%	8%	9%	10%
35%	8%	10%	11%	12%
40%	10%	12%	13%	14%
45%	12%	14%	15%	17%
50%	14%	17%	18%	19%
55%	15%	19%	20%	22%
60%	18%	22%	23%	25%
65%	20%	24%	26%	28%
70%	23%	28%	30%	32%
75%	25%	31%	34%	36%
80%	29%	35%	38%	40%
85%	32%	39%	42%	45%
90%	36%	44%	48%	51%
95%	42%	51%	54%	58%

Supplementary References

1. Brooks, T. M. *et al.* Measuring terrestrial Area of Habitat (AOH) and its utility for the IUCN Red List. *Trends in Ecology & Evolution* **34**, 977–986 (2019).
2. Hanson, J. O. *et al.* Global conservation of species' niches. *Nature* **580**, 232–234 (2020).
3. Rodrigues, A. S. L. *et al.* Global gap analysis: Priority regions for expanding the global protected-area network. *BioScience* **54**, 1092–1100 (2004).
4. Schuster, R. *et al.* Optimizing the conservation of migratory species over their full annual cycle. *Nature Communications* **10**, 1754 (2019).
5. Riggio, J. *et al.* Global human influence maps reveal clear opportunities in conserving Earth's remaining intact terrestrial ecosystems. *Global Change Biology* **26**, 4344–4356 (2020).
6. Harris, T., Mulligan, M. & Brummitt, N. Opportunities and challenges for herbaria in studying the spatial variation in plant functional diversity. *Systematics and Biodiversity* **19**, 322–332 (2021).
7. Sayre, R. *et al.* An assessment of the representation of ecosystems in global protected areas using new maps of World Climate Regions and World Ecosystems. *Global Ecology and Conservation* **21**, e00860 (2020).
8. Rose, A. N., McKee, J. J., Urban, M. L. & Bright, E. A. LandScan 2017. (2018).
9. Zhou, Y., Varquez, A. C. G. & Kanda, M. High-resolution global urban growth projection based on multiple applications of the SLEUTH urban growth model. *Scientific Data* **6**, 34 (2019).
10. Schneider, A., Friedl, M. A. & Potere, D. A new map of global urban extent from MODIS satellite data. *Environ. Res. Lett.* **4**, 044003 (2009).

11. Oakleaf, J. R. *et al.* Mapping global development potential for renewable energy, fossil fuels, mining and agriculture sectors. *Scientific Data* **6**, 101 (2019).
12. Johnson, J. A. *et al.* Energy matters: Mitigating the impacts of future land expansion will require managing energy and extractive footprints. *Ecological Economics* **187**, 107106 (2021).
13. Hewson, J., Crema, S. C., González-Roglich, M., Tabor, K. & Harvey, C. A. New 1 km Resolution Datasets of Global and Regional Risks of Tree Cover Loss. *Land* **8**, 14 (2019).
14. Esri, Clark Labs, & ESA CCI. Converted Lands 2018 to 2050. (2021).
15. Hewson, J., Crema, S., González-Roglich, M., Tabor, K. & Harvey, C. Global model results for transition potential. (2019) doi:10.5281/ZENODO.3237796.
16. Chaplin-Kramer, R. *et al.* Mapping the planet's critical natural assets. *Nat Ecol Evol* **7**, 51–61 (2022).
17. ESA Climate Change Initiative - Land Cover project. *Land Cover CCI*. https://maps.elie.ucl.ac.be/CCI/viewer/download.php (2017).
18. UNEP-WCMC and IUCN. *Protected Planet: The World Database on Protected Areas (WDPA)*. https://www.protectedplanet.net (2021).
19. Chaplin-Kramer, R. *et al.* Critical Natural Assets. (2020) doi:10.17605/OSF.IO/R5XZ7.
20. Mulligan, M. *Documentation for the Co$tingNature Model V3*. https://docs.google.com/document/d/136OvAO6PSyVBp0gNl9f0_pIAIg-h-4JZGArnf2V6-Us/edit?usp=embed_facebook (2018).
21. Gunnell, K., Mulligan, M., Francis, R. A. & Hole, D. G. Evaluating natural infrastructure for flood management within the watersheds of selected global cities. *Science of The Total Environment* **670**, 411–424 (2019).

22. Mulligan, M. *WaterWorld (AguAAndes) v2.x Model Documentation*. https://docs.google.com/document/d/1GKheQFp5_rsZyazwCJxCzeEStQF2jh04x-Dl_oG5yoY/edit (2022).

23. Noon, M. L. *et al.* Mapping the irrecoverable carbon in Earth's ecosystems. *Nat Sustain* **5**, 37–46 (2022).

24. Noon, M. *et al.* Mapping the irrecoverable carbon in Earth's ecosystems. (2021) doi:10.5281/ZENODO.4091029.

25. Neugarten, R. A. *et al.* Mapping the planet's critical areas for biodiversity and people. (2022) doi:10.5281/ZENODO.7853188.

26. Esri. World Countries. https://www.arcgis.com/home/item.html?id=d974d9c6bc924ae0a2ffea0a46d71e3d (2020).

27. Dinerstein, E. *et al.* An ecoregion-based approach to protecting half the terrestrial realm. *BioScience* **67**, 534–545 (2017).
