# Supplementary material for: Mapping the planet’s critical areas for biodiversity and nature’s contributions to people
Source: Nat Commun. 2024 Jan 10;15:261. doi: 10.1038/s41467-023-43832-9 (PMC10781687; doi:10.1038/s41467-023-43832-9)
Supplement: Supplementary file 3 — Description of Additional Supplementary Files [file 41467_2023_43832_MOESM3_ESM.pdf]

Description of Additional Supplementary Files

Supplementary Data 1. Prioritized areas for NCP and biodiversity; results disaggregated by country, continent and biome.

Supplementary Data 2. Prioritized areas for NCP and biodiversity combined with areas of high development potential; results disaggregated by country, continent, and biome.
