# Supplementary material for: Mapping the planet’s critical areas for biodiversity and nature’s contributions to people
Source: Nat Commun. 2024 Jan 10;15:261. doi: 10.1038/s41467-023-43832-9 (PMC10781687; doi:10.1038/s41467-023-43832-9)
Supplement: Supplementary file 6 — Reporting Summary [file 41467_2023_43832_MOESM6_ESM.pdf]

nature portfolio

Corresponding author(s): Rachel A Neugarten

Last updated by author(s): Oct 29, 2023

Reporting Summary

Nature Portfolio wishes to improve the reproducibility of the work that we publish. This form provides structure for consistency and transparency in reporting. For further information on Nature Portfolio policies, see our Editorial Policies and the Editorial Policy Checklist.

Statistics

For all statistical analyses, confirm that the following items are present in the figure legend, table legend, main text, or Methods section.

n/a	Confirmed	
x	☐	The exact sample size (*n*) for each experimental group/condition, given as a discrete number and unit of measurement
x	☐	A statement on whether measurements were taken from distinct samples or whether the same sample was measured repeatedly
x	☐	The statistical test(s) used AND whether they are one- or two-sided<br>*Only common tests should be described solely by name; describe more complex techniques in the Methods section.*
x	☐	A description of all covariates tested
x	☐	A description of any assumptions or corrections, such as tests of normality and adjustment for multiple comparisons
x	☐	A full description of the statistical parameters including central tendency (e.g. means) or other basic estimates (e.g. regression coefficient) AND variation (e.g. standard deviation) or associated estimates of uncertainty (e.g. confidence intervals)
x	☐	For null hypothesis testing, the test statistic (e.g. *F*, *t*, *r*) with confidence intervals, effect sizes, degrees of freedom and *P* value noted<br>*Give P values as exact values whenever suitable.*
x	☐	For Bayesian analysis, information on the choice of priors and Markov chain Monte Carlo settings
x	☐	For hierarchical and complex designs, identification of the appropriate level for tests and full reporting of outcomes
x	☐	Estimates of effect sizes (e.g. Cohen's *d*, Pearson's *r*), indicating how they were calculated

Our web collection on statistics for biologists contains articles on many of the points above.

Software and code

Policy information about availability of computer code

Data collection	No software was used in data collection.
Data analysis	Spatial optimizations were conducted using the prioritizr R package (version 8.0.3, https://prioritizr.net), Gurobi (version 9.1.2 https://www.gurobi.com), and R (version 4.2.2, https://cran.r-project.org/bin/windows/base/). Code is available on Zenodo: https://zenodo.org/record/8225989 Spatial analyses other than optimizations were conducted using QGIS (version 3.26.3) and ArcGIS Desktop (version 10.7).

For manuscripts utilizing custom algorithms or software that are central to the research but not yet described in published literature, software must be made available to editors and reviewers. We strongly encourage code deposition in a community repository (e.g. GitHub). See the Nature Portfolio guidelines for submitting code & software for further information.

Data

Policy information about availability of data

All manuscripts must include a data availability statement. This statement should provide the following information, where applicable:

- Accession codes, unique identifiers, or web links for publicly available datasets
- A description of any restrictions on data availability
- For clinical datasets or third party data, please ensure that the statement adheres to our policy

The data on prioritized areas and high development potential areas generated in this study have been deposited in the Zenodo database under accession code 10.5281/zenodo.7853188 [https://zenodo.org/records/7853188]. The prioritized areas data disaggregated by country, continent, and biome generated in this study are provided in the Supplementary Data files 1 and 2. Data on nature's contributions to people used in this study are available in the Open Science Framework

database under accession code 10.17605/OSF.IO/R5XZ7 [https://osf.io/r5xz7/] and can be visualized at https://bit.ly/3Jk8vDo. The biodiversity data used in this study are available under restricted access for non-commercial use, access can be obtained by request [https://www.iucnredlist.org/resources/spatial-data-download]. The WDPA and OECM data used in this study are available under restricted access for non-commercial use, access can be obtained by request [www.protectedplanet.net]. The vulnerable carbon data used in this study are available in the Zenodo database under accession code 10.5281/zenodo.4091029 [https://zenodo.org/records/4091029]. The data on projected tree cover loss used in this study are available in the Zenodo database under accession code 10.5281/zenodo.3237796 [https://zenodo.org/records/3237796]. The data on areas vulnerable to land cover change used in this study are available from ArcGIS Online [https://www.arcgis.com/home/item.html?id=645c280931ac486cadb92c828eac09e3].

Research involving human participants, their data, or biological material

Policy information about studies with human participants or human data. See also policy information about sex, gender (identity/presentation), and sexual orientation and race, ethnicity and racism.

Reporting on sex and gender	NA
Reporting on race, ethnicity, or other socially relevant groupings	NA
Population characteristics	NA
Recruitment	NA
Ethics oversight	NA

Note that full information on the approval of the study protocol must also be provided in the manuscript.

Field-specific reporting

Please select the one below that is the best fit for your research. If you are not sure, read the appropriate sections before making your selection.

☐ Life sciences ☐ Behavioural & social sciences ☒ Ecological, evolutionary & environmental sciences

For a reference copy of the document with all sections, see nature.com/documents/nr-reporting-summary-flat.pdf

Ecological, evolutionary & environmental sciences study design

All studies must disclose on these points even when the disclosure is negative.

Study description	We conducted spatial optimization to identify globally important places for nature's contributions to people and biodiversity, and calculated areas of overlap with areas of high potential for development, using published data sets and open source software. This is a non-experimental analysis (spatial analysis) and therefore has no research sample, sampling strategy, randomization, nor blinding. The spatial scale is global. The timing of the secondary datasets used as inputs from the analysis spans 2000-2017.
Research sample	No sampling was performed.
Sampling strategy	No sampling was performed.
Data collection	No data collection was performed, our analysis relies on secondary data and spatial modeling.
Timing and spatial scale	The spatial scale is global. Secondary spatial datasets used as inputs to our analysis span a 2000-2017 window.
Data exclusions	No data were excluded.
Reproducibility	Code is published to allow reproducibility.
Randomization	No randomization (no sampling nor experiment was performed.)
Blinding	No experiment was performed, not applicable.

Did the study involve field work? ☐ Yes ☒ No

Reporting for specific materials, systems and methods

We require information from authors about some types of materials, experimental systems and methods used in many studies. Here, indicate whether each material, system or method listed is relevant to your study. If you are not sure if a list item applies to your research, read the appropriate section before selecting a response.

Materials & experimental systems

n/a	Involved in the study
☒	☐ Antibodies
☒	☐ Eukaryotic cell lines
☒	☐ Palaeontology and archaeology
☒	☐ Animals and other organisms
☒	☐ Clinical data
☒	☐ Dual use research of concern
☒	☐ Plants

Methods

n/a	Involved in the study
☒	☐ ChIP-seq
☒	☐ Flow cytometry
☒	☐ MRI-based neuroimaging
